# Personality and Developmental Characteristics of Primary School Students**’** Personality Types

**DOI:** 10.3389/fpsyg.2021.693329

**Published:** 2021-08-18

**Authors:** Yongjin Yu, Yanyan Zhang

**Affiliations:** Department of Psychology, School of Philosophy and Sociology, Jilin University, Changchun, China

**Keywords:** primary school students, personality types, latent profile analysis, personality characteristic, developmental characteristic

## Abstract

The aim of the current study was to investigate the personality characteristics and developmental characteristics of primary school students’ personality types in a cross-sectional sample of 10,366 Chinese children. The *Personality Inventory for Primary School Student* was used to evaluate primary school students’ personality. Latent profile analysis (LPA) was used to classify primary school students’ personality types. One-way ANOVA was used to explore the personality characteristics of personality types, and Chi-square tests were used to investigate grade and gender differences of primary school students’ personality types. Results showed that the primary school students could be divided into three personality types: the resilient, the overcontrolled, and the undercontrolled. Resilients had the highest scores, and undercontrollers had the lowest scores on all of five personality dimensions (intelligence, conscientiousness, extraversion, agreeableness, and emotional stability). The overcontrollers’ scores on personality were between the other two types, with lower emotional stability. As the grade level increased, the proportion of undercontrolled students in primary schools generally showed an upward trend and reached the maximum in grade 5. The proportion of resilient students in primary schools generally showed a downward trend. The proportion of resilient students was highest in grade 2 and lowest in grade 5. Girls were significantly more likely than boys to be resilient personality types, while boys were significantly more likely than girls to be undercontrolled personality types. The overcontrolled personality type did not show significant gender differences. Because of the undesirable internalizing problems related to overcontrollers and the externalizing problems related to undercontrollers, our results have implications for Chinese schools, families, and society in general.

## Introduction

Personality has significant impacts on many aspects of people’s everyday lives, such as interpersonal relationships, health, academic performance, and subjective wellbeing (Neyer et al., 2014; Briki, 2018; Gray and Pinchot, 2018; Stajkovic et al., 2018). The primary school stage is an important developmental period for accumulating knowledge and learning to understand society. It is also an important stage for children’s personality development (Soto and Tackett, 2015). Previous research has shown that personality development in primary school can effectively predict crime in adulthood (Kachaeva et al., 2017). In studies of personality development, two main strategies a variable-centered and a person-centered approach are being distinguished (Donnellan and Robins, 2010).

Variable-centered approaches are primarily reflected in studies on personality dimensions or traits, such as the dimensions of the five-factor model (FFM) of personality, the most widely employed model (Bergman and Magnusson, 1997; John et al., 2008). The FFM yields five personality dimensions: agreeableness, extraversion, conscientiousness, emotional stability, and openness. Personality is the psychological properties with culture attribute (Li and Zhang, 2006; Zhang, 2011). Based on Chinese culture and the characteristics of children’s personalities, Yang (2014) used teachers’ free description and vocabulary to collect and code personality trait adjectives for primary school students in China. They decided that the personality construction of Chinese primary school students was composed of five dimensions: extraversion, agreeableness, conscientiousness, emotional stability, and intelligence. Intelligence refers to the characteristics of individual self-awareness, intelligence, and talents, reflecting whether primary school students have their own independent ideas, high learning ability, and positive motivation in learning activities. Teachers’ assessment of intelligence was reflected in three aspects (intelligent, curiosity/creativity, and independent/enterprising). Western countries named the traits related to intelligence as openness. In addition to emphasizing the speed of brain reaction, it also emphasized appreciation of personal emotion, imagination, and the pursuit of a better life, beliefs, and values (Carver and Scheier, 1996). In China, this dimension mainly reflects the speed of children’s brain reaction. This dimension also includes children’s independence and enterprising spirit (Zhang, 2011). The dimension named as intelligence is more in line with Chinese educational philosophy. Cultural differences lead to differences in the connotations of personality traits involving intelligence.

Person-centered approaches study “types” identifying clusters of individuals with similar personality patterns (Van Leeuwen et al., 2004). Person-centered approaches are concerned with how different dimensions are organized within the individual, which subsequently defines different types of person (Herzberg and Roth, 2006). The typological approach emphasizes persons (Hart et al., 2003). Personality types are intended specifically to represent the organization among traits that occurs within individuals (Grumm and Collani, 2009). According to the results of several studies, the five personality traits in FFM can be combined with each other to form three personality types, resilient, undercontrolled, and overcontrolled (Robins et al., 1996; Asendorpf and Van Aken, 1999; Asendorpf et al., 2001; Yang and Ma, 2014; Rosenström and Jokela, 2017). These three personality types have been repeatedly verified across different languages and cultures, different personality models, and different ages (Donnellan and Robins, 2010; Chapman and Goldberg, 2011; Meeus et al., 2011; Alessandri et al., 2014; Leikas and Salmela-Aro, 2014; Specht et al., 2014; Yang and Ma, 2014). Previous research described the three personality types in terms of the FFM of personality description. Resilients were characterized by relatively high levels of openness, conscientiousness, extraversion, emotional stability, and agreeableness. Overcontrollers were characterized by low emotional stability, with moderate levels of the other four dimensions. Undercontrollers were characterized by relatively low levels of openness, conscientiousness, extraversion, emotional stability, and agreeableness (Grumm and Collani, 2009; Zentner and Shiner, 2012; Yang and Ma, 2014; Chen, 2019; Zou et al., 2019). The resilient personality type is well adapted to society, verbally expressive, energetic, independent, self-confident, and able to adjust to situational demands using self-control. The overcontrolled personality type is socially maladaptive, emotionally brittle, interpersonally sensitive, tense, and inhibited prone to excessively restraining impulses. The undercontrolled personality type is socially maladaptive, impulsive, self-centered, manipulative, confrontational, disagreeable, and lacking in self-control (Block and Block, 1980; Robins et al., 1996; Donnellan and Robins, 2010).

The identification of groups of persons is frequently done through cluster analysis (e.g., Magnusson and Bergman, 1990) or Q factor analysis (e.g., York and John, 1992). The goal of these analytic techniques is to maximize similarity among members of a group while minimizing resemblance of members of one group to members of all the others. These approaches have methodological limitations (Klimstra et al., 2010). The number of types determined by these analytic techniques has the subjective judgment and theoretical orientation of the investigators. Moreover, the number of groups that is specified has implications for statistical analysis: If many groups are specified, then there may be few participants in each group, which makes parametric data analysis difficult. On the other hand, the formation of only a few groups may mean that participants have only limited resemblance to other participants in the same group, and consequently, viewing this group as representing a type of person can be misleading. There are no easy resolutions of this problem, which has led to calls for the development of new analytic techniques for person-centered research (Singer and Ryff, 2001). LPA is an empirically driven method that defines taxonomies or classes of people based on common characteristics (Lanza et al., 2003). LPA uses all observations of the continuous dependent variable to define these classes *via* maximum likelihood estimation (Little and Rubin, 1987). Some studies point out that LPA is better than traditional Q factor analysis and cluster analysis (Reinke et al., 2008). It not only eliminates the measurement errors in the construct, but also provides the researcher with more objective indices of fit to make up for the deficiencies of Q factor analysis and cluster analysis (Bauer and Curran, 2004). Meeus et al. (2011) used LPA to divide adolescents into three personality types: the resilient, the overcontrolled, and the undercontrolled. This study found that as the age increased, the number of overcontrollers and undercontrollers decreased, whereas the number of resilients increased. Undercontrollers, in particular, were found to peak in early to middle adolescence. Asendorpf and van Aken (1999) classified the personality types of children from 4 to 10 years old, and found that more girls were rated as the resilient type than boys, and less girls were rated as the undercontrolled type than boys. However, in the context of Chinese culture, there are few studies on the personality characteristics and developmental characteristics of primary school students’ personality types.

Asendorpf and van Aken (1999) found that childhood personality types are good predictors of later development. Adjustment problems differ by personality type, which indicates the utility of conceptualizing students’ personalities in terms of types for both research and clinical practice (Shiner and Caspi, 2003). Overcontrolled children may be especially at risk of developing internalizing problems (e.g., symptoms of depression and anxiety) due to their inhibited nature, whereas undercontrollers’ impulsivity may leave them vulnerable to the development of externalizing problems (e.g., aggression and attention problems; Yu et al., 2015; Achenbach et al., 2016; Bohane et al., 2017). Robins et al. (1996) found that undercontrollers had lower IQ scores, lower academic achievement at school, worse conduct, and more serious delinquency than overcontrollers and resilients. Focusing on personality types allows us to discern predictable patterns of risks to healthy development, helping teachers and parents educate children and intervene when necessary. Teachers and parents are more likely to prevent child maladaptive development based on developmental characteristics. Our research lays the foundation for psychologists to conduct future intervention research.

The current study aimed to investigate the personality characteristics of Chinese primary school students’ personality types and their developmental characteristics by grade and gender. Based on previous research, we hypothesized that (1) the primary school students would be divided into three personality types: the resilient, the overcontrolled, and the undercontrolled; (2) compared to the other two types, undercontrollers’ scores would be lower and resilients’ would be higher on all five personality dimensions (intelligence, conscientiousness, extraversion, agreeableness, and emotional stability), with the overcontrollers’ scores in between, with lower emotional stability; and (3) there would be significant grade and gender differences in primary school students’ personality types.

## Materials and Methods

### Participants and Procedures

We selected 21 primary schools in North China to issue the questionnaire. The questionnaire was used to evaluate primary school students by teachers. There were several classes for each grade and two teachers in each class. We took a multi-informant approach to reduce reporter bias in measurement of personality. Each student was assessed by two teachers; 636 teachers rated 10,366 primary school students (5,441 male and 4,925 female). There were 2,209 first graders, with an average age of 6.92 years old, 2,066 second graders, average age 8.06 years old, 2,218 third graders, average age 9.41 years old, 1,886 fourth graders, average age 10.03 years old, and 2,087 fifth graders, average age 11.24 years old. Written informed consent had been obtained from the parents’ guardians of all participants. All participants volunteered to join the experiments, and informed consents signed by their legal guardians.

### Measures

#### Personality Inventory for Primary School Student

Teachers rated their students’ personality on the Personality Inventory for Primary School Student (Zhang, 2011). The personality was measured using Zhang’s Chinese FFM, which was developed based on the original FFM. This personality inventory includes five dimensions, namely, extraversion, agreeableness, conscientiousness, emotional stability, and intelligence. This inventory includes 62 items. All items were rated on a five-point Likert scale from 1 (very inaccurate) to 5 (very accurate). Emotional stability is an inverted dimension. Items rated as 1 point are converted into 5 points, and items rated as 2 points are converted into 4 points. For example, when criticized by the teacher, the student will immediately become angry or frustrated. This item is a reverse scoring. After reverse scoring, the total score of each personality dimension is calculated. The higher the score, the higher the development level of the personality dimension. This questionnaire has good reliability and validity in the Chinese cultural context (Zhang, 2011). In order to investigate the degree of consistency of the teacher’s evaluations, we had two teachers in each class fill out the personality rating scale on all primary school students in the class at the same time. The rater reliability and the Cronbach’s alphas of scale are presented in Table 1. The consistency of the two raters’ evaluations of primary school students indicated that the evaluation results were objective and credible.

**Table 1:** The rater reliability and the Cronbach’s alphas of the scale.

	Intelligence	Conscientiousness	Extraversion	Agreeableness	Emotional stability
Cronbach’s α	0.958	0.946	0.915	0.945	0.863
Rater reliability	0.888	0.887	0.876	0.872	0.814

### Data Analysis

The SPSS 20.0 was used to conduct descriptive statistics and analysis of variance. Specifically, One-way ANOVA was used to explore the personality characteristics of personality types, and Chi-square tests were used to investigate the grade and gender differences of primary school students’ personality types. The Mplus 7.4 was used to conduct LPA. All data were treated with a statistical significance level of *p* < 0.05. LPA was executed to analyze primary school students’ personality types (Muthén and Muthén, 2000). We chose the optimal model relying on the following criteria: Akaïke Information Criterion (AIC), Bayesian Information Criterion (BIC), Sample-Size Adjusted Bayesian Information Criterion (aBIC), Likelihood Ratio Tests (LMR), Bootstrap Likelihood Ratio Test (BLRT), and Entropy (Nylund et al., 2007). Smaller values of AIC, BIC, and aBIC indicate better models (Schwarz, 1978). The range of entropy is between 0.00 and 1.00. Higher values of entropy indicate higher the classification accuracy (Hix-Small et al., 2004). For the LMR and BLRT, a value of *p* smaller than 0.05 suggests that the k class model is better than the k-1 class model (Asparouhov and Muthén, 2012). If some types in a k class model already appear in a k-1 class model, the k-1 class model will be selected according to the principle of model simplicity (Muthén and Muthén, 2000). In order to make the model more universal, Fisher and Robie (2019) pointed out that the large sample size should be randomly divided into two small samples, and LPA should be done separately. The SPSS 20.0 was used to randomly split a large sample size into two small subsamples. One subsample was used for exploratory LPA. Another subsample was used for cross-validation.

## Results

### Personality Characteristics of Primary School Students’ Personality Types

**Figure 1:**
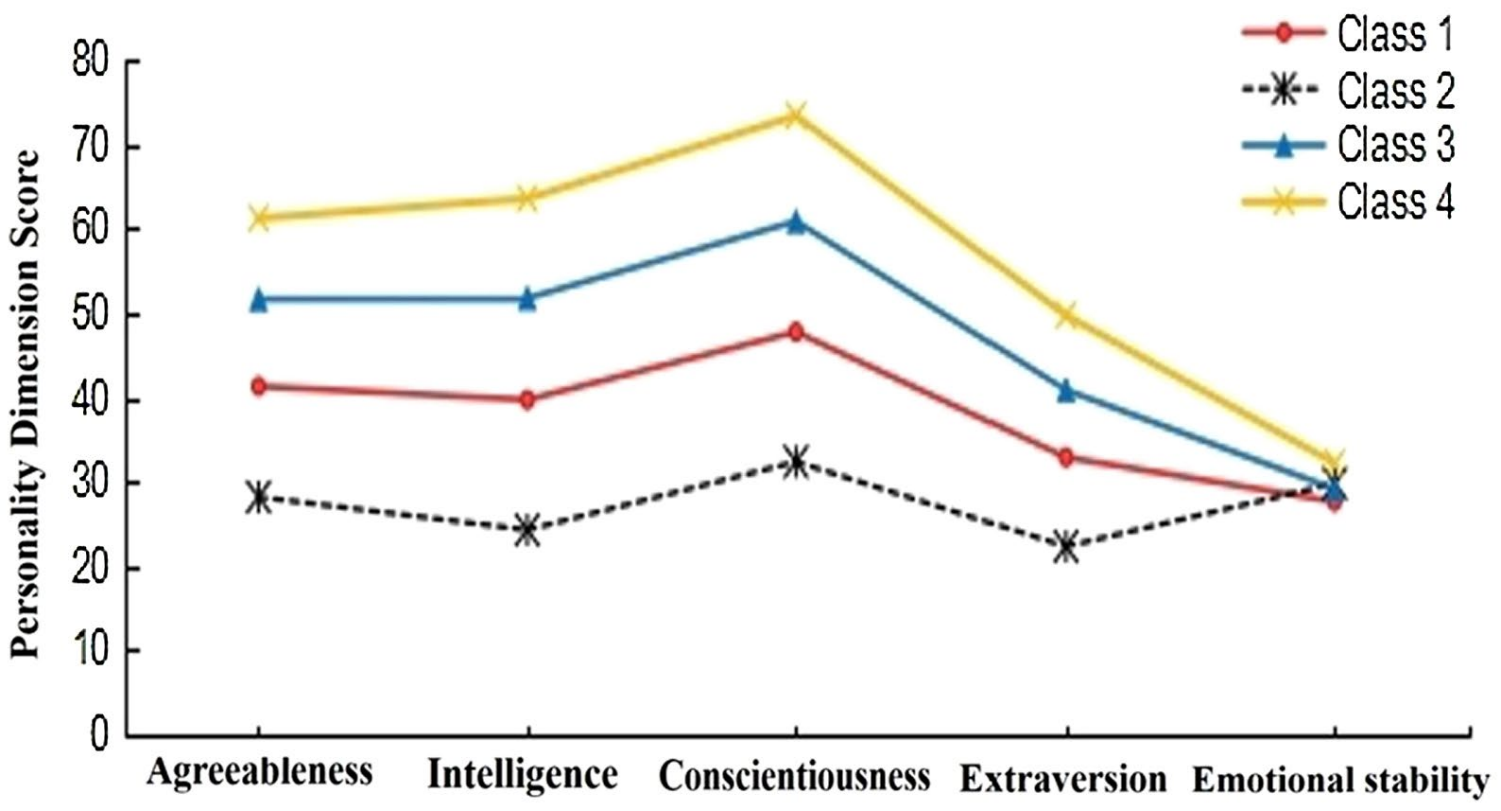
Potential profile of the four-class model (*n*1 = 5,183).

**Figure 2:**
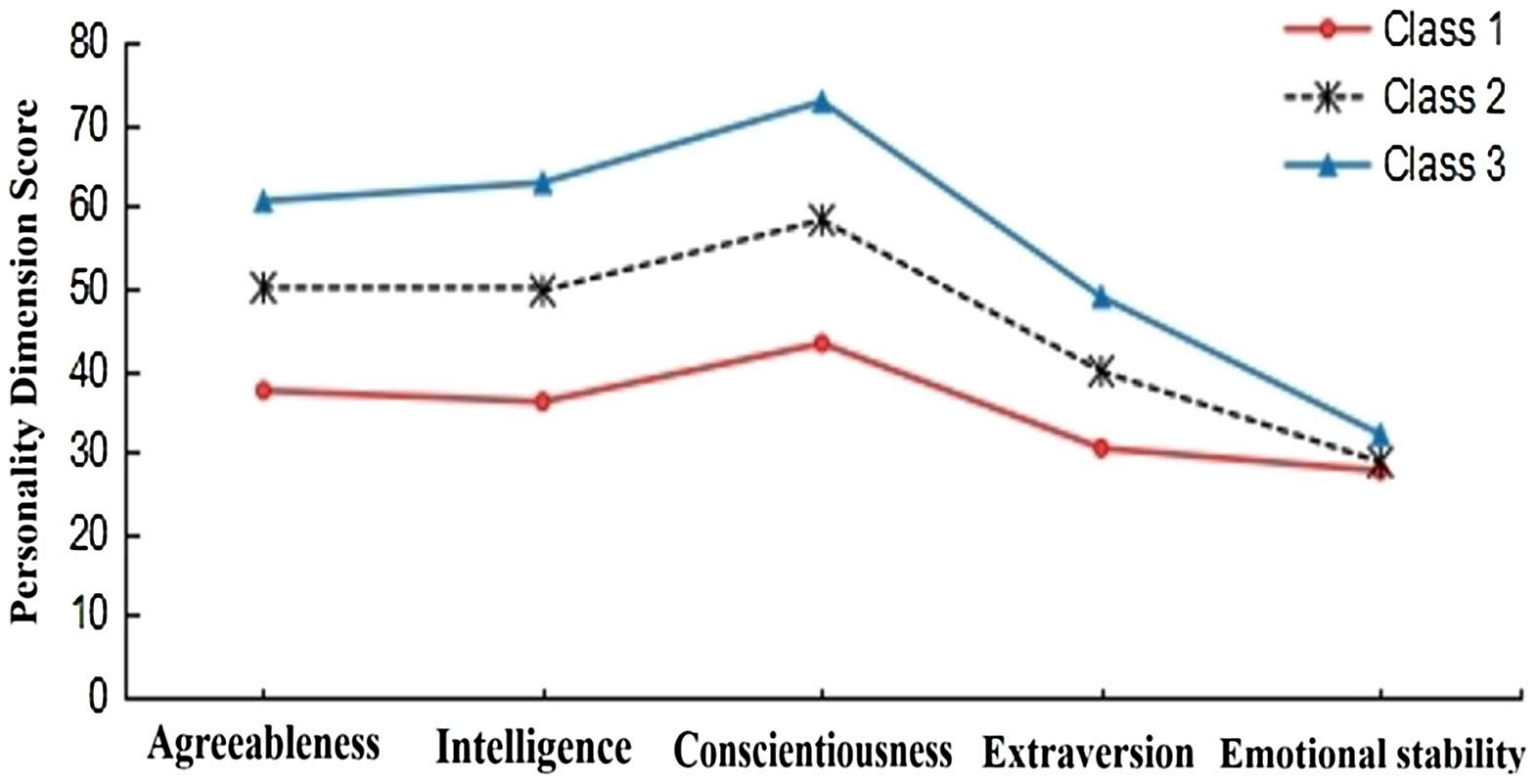
Potential profile of the three-class model (*n*1 = 5183).

**Figure 3:**
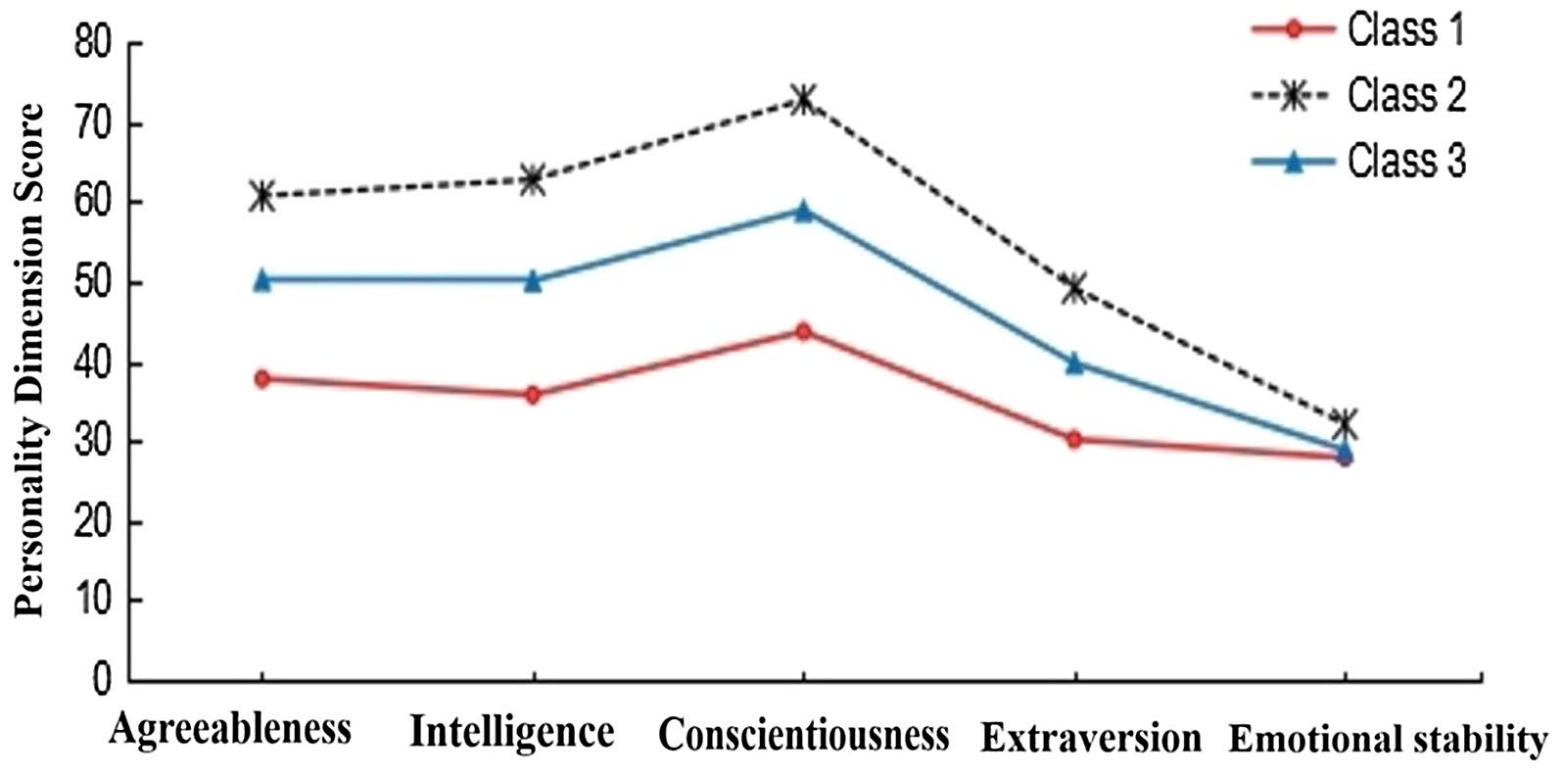
Potential profile of the three-class model (*n*2 = 5,183).

A total of 10,366 participants were randomly divided into two samples. Sample 1 (*n*1 = 5,183) and sample 2 (*n*2 = 5,183) were used for LPA, respectively. The results showed that the potential category classifications of the two samples were similar (see Table 2). AIC, BIC, and adjusted BIC of the three-class model and four-class model were smaller than other models, the entropy values were all above 0.8, and the value of *p* for LMR and BLRT was both significant, which indicates that these two models fit well and the correct rate of personality type classification is higher than other models. Wang and Bi (2018) pointed out that the final model should be determined in conjunction with the actual meaning of classification. In the four-class model, the characteristics of the two types in the middle overlap and should be regarded as one type. In other words, the personality characteristics of these two types were very similar (see Figure 1). According to the principle of model simplicity, the three-class model should be the optimal model. In addition, the four-class model did not meet the condition that each type accounts for at least 5% of the total sample (Nagin, 2005). The three-class model has clearer and more concise outlines, and the indicators also meet the criteria for suitability of LPA. LPA diagrams of the two samples are presented in Figures 2, 3. The results showed that the three-class model was the best model. It is reasonable to divide the personality of primary school students into three classes. This result is consistent with the number of types classified by Ma (2016).

**Table 2:** Latent profile analysis fitting index of personality dimension of primary school students (*n* = 10,366).

Number of types	AIC	BIC	aBIC	Entropy	*p*(LMR)	*p*(BLRT)	Category probability
Sample 1							
One type	192450.362	192515.893	192484.116				1
Two types	182102.152	182207.002	182156.159	0.857	0.000	0.000	0.450/0.550
Three types	178131.770	178275.939	178206.030	0.854	0.000	0.000	0.274/0.439/0.287
Four types	175075.072	175258.560	175169.586	0.892	0.000	0.000	0.307/ 0.047/ 0.391/ 0.255
Sample 2							
One type	192475.752	192541.284	192509.507				1
Two types	182225.194	182330.045	182279.202	0.862	0.000	0.000	0.442/0.558
Three types	178432.050	178576.219	178506.310	0.852	0.000	0.000	0.282/0.272/0.446
Four types	175408.143	175591.631	175502.656	0.884	0.000	0.000	0.308/0.049/0.400/0.243

One-way ANOVA and multiple comparisons were used to describe the characteristics of each personality type (see Table 3). The third personality type had the highest scores on all five dimensions (intelligence, conscientiousness, extraversion, emotional stability, and agreeableness). According to Robins et al. (1996), we identified this type as the resilient personality type. The score of the second type on personality was between the other two types, with lower emotional stability. According to Zentner and Shiner (2012), we identified this type as the overcontrolled personality type. The first personality type had the lowest scores on all five dimensions. According to Ma (2016), we identified this type as the undercontrolled personality type.

**Table 3:** Descriptive statistics, analysis of variance, and *post-hoc* tests on personality types in five dimensions.

Personality types	*N*	Intelligence	Conscientiousness	Extraversion	Agreeableness	Emotional stability
1	1422	36.14 ± 8.51	43.27 ± 8.46	30.45 ± 6.27	37.50 ± 6.87	27.67 ± 5.19
2	2272	49.71 ± 6.53	58.37 ± 6.91	39.86 ± 5.09	50.09 ± 4.98	28.75 ± 6.43
3	1489	62.94 ± 5.61	72.87 ± 5.54	48.97 ± 4.47	60.69 ± 3.95	32.14 ± 7.63
*F*_(2,5,180)_		5488.666^***^	6453.527^***^	4469.464^***^	6925.730^***^	194.254^***^
The *post-hoc test*		1 < 2 < 3	1 < 2 < 3	1 < 2 < 3	1 < 2 < 3	1 < 2 < 3
*Partial η* ^2^		0.679	0.714	0.633	0.728	0.070

*<, significantly lower; =, no significant difference; and >, significantly higher*.

*** *p* < 0.001;

^**^p < 0.01; ^*^p < 0.05.

### Developmental Characteristics of Primary School Students’ Personality Types

**Figure 4:**
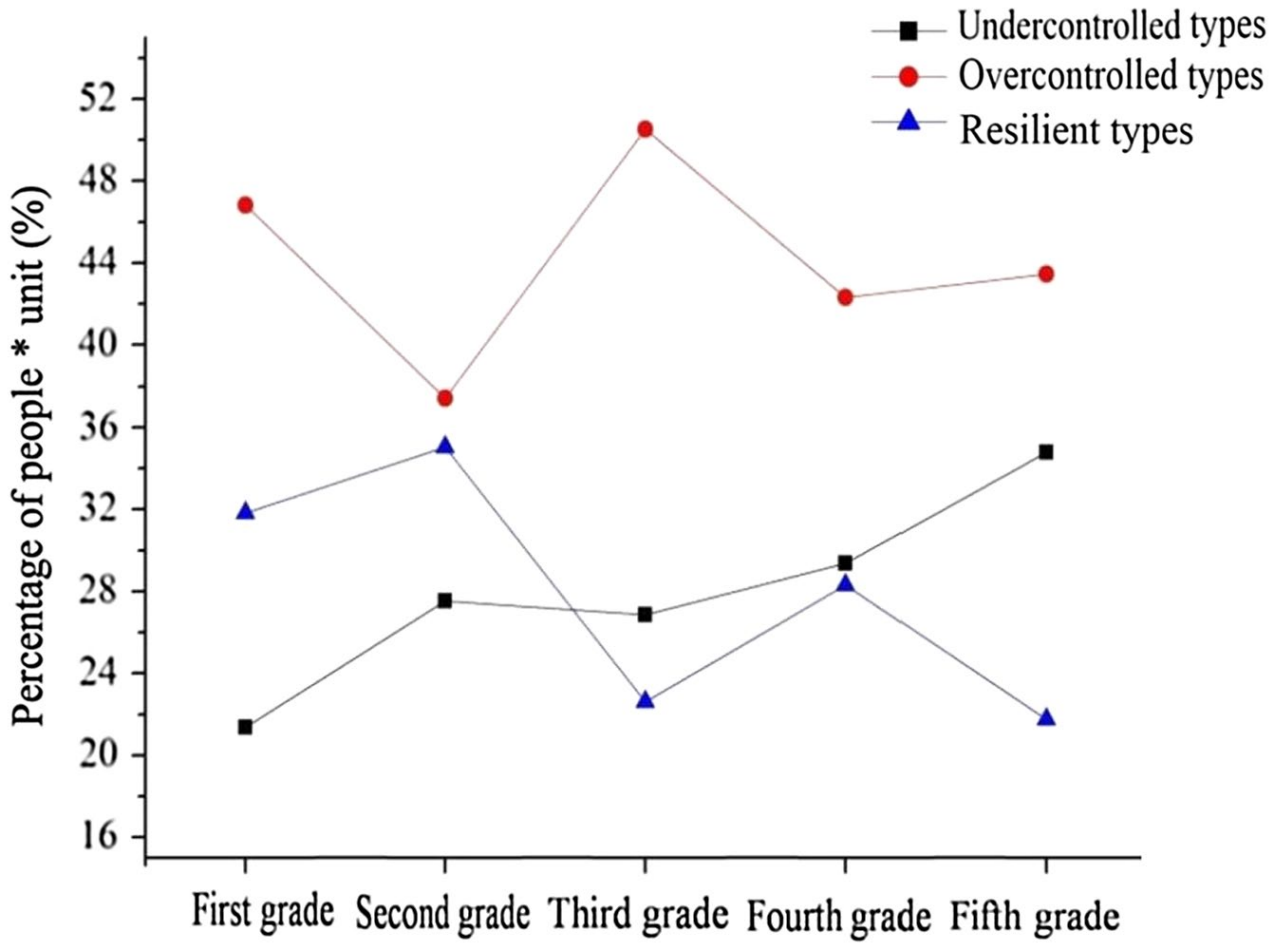
Developmental trends of primary school students’ personality types.

In order to investigate the grade and gender differences in primary school students’ personality types, we first confirmed that the personality types were related to gender and grade (see Table 4). Results showed that personality types were indeed related to both grade (*χ*^2^_(8)_ = 217.016^**^, *Φ* = 0.102) and gender (*χ*^2^_(2)_ = 141.368^**^, *Φ* = 0.117). Since the interaction effects between grade and gender had no significant influence on personality type, we only examined the relationship between gender and personality type, and the relationship between grade and personality type. The second step was to examine the developmental characteristics of primary school students’ personality types in different grades. The population proportions presented in Figure 4 show the grade-related developmental trajectory of primary school students’ personality types. Chi-square tests were used to examine grade differences. Finally, a Chi-square test was conducted to examine gender differences.

**Table 4:** The number and ratio of each type of personality in each grade and gender.

	First grade	Second grade	Third grade	Fourth grade	Fifth grade	Girl	Boy
Undercontroller (*n* = 2890)	472 (21.4%)	569 (27.5%)	569 (26.9%)	554 (29.4%)	726 (34.8%)	1136 (23.1%)	1754 (32.2%)
Overcontroller (*n* = 4582)	1034 (46.8%)	773 (37.4%)	1070 (50.5%)	798 (42.3%)	907 (43.4%)	2201 (44.7%)	2381 (43.8%)
Resilient (*n* = 2894)	703 (31.8%)	724 (35.1%)	479 (22.6%)	534 (28.3%)	454 (21.8%)	1588 (32.2%)	1306 (24.0%)
Total (*n* = 10366)	2209 (100%)	2066 (100%)	2118 (100%)	1886 (100%)	2087 (100%)	4925 (100%)	5441 (100%)

Results showed that among the undercontrolled primary school students, there were significant differences between different grades (*χ*^2^_(4)_ = 99.413^**^, *Φ* = 0.098). The proportion of students in grade 1 was significantly lower than grade 2 (*χ*^2^_(1)_ = 22.091^**^, *Φ* = 0.072), grade 3 (*χ*^2^_(1)_ = 17.888^**^, *Φ* = 0.064), grade 4 (*χ*^2^_(1)_ = 34.738^**^, *Φ* = 0.092), and grade 5 (*χ*^2^_(1)_ = 96.101^**^, *Φ* = 0.150). There was no significant difference in the proportion between grade 2 and grade 3 (*χ*^2^_(1)_ = 0.241, *Φ* = 0.008). There was no significant difference in the proportion between grade 2 and grade 4 (*χ*^2^_(1)_ = 1.629, *Φ* = 0.020). The proportion of students in grade 2 was significantly lower than that in grade 5 (*χ*^2^_(1)_ = 25.400^**^, *Φ* = 0.078). The proportion of students in grade 3 was significantly lower than grade 4 (*χ*^2^_(1)_ = 3.113^△^, *Φ* = 0.028) and grade 5 (*χ*^2^_(1)_ = 30.953^**^, *Φ* = 0.086). The proportion of students in grade 4 was significantly lower than grade 5 (*χ*^2^_(1)_ = 13.290^**^, *Φ* = 0.058). This result showed that as the grade level increased, the proportion of undercontrolled students in primary schools generally showed an upward trend. Specifically, this type showed an upward trend from grade 1 to grade 2, a flat trend from grade 2 to grade 3, and an upward trend from grade 3 to grade 5. The proportion of boys in the undercontrolled primary school students was significantly higher than that of the girls (*χ*^2^_(1)_ = 108.128^**^, *Φ* = 0.102). Our results revealed a significant gender difference in the undercontrolled primary school students.

Among the overcontrolled primary school students, there were significant differences between different grades (*χ*^2^_(4)_ = 82.138^**^, *Φ* = 0.089). The proportion of students in grade 1 was significantly higher than grade 2 (*χ*^2^_(1)_ = 38.600^**^, *Φ* = 0.095), grade 4 (*χ*^2^_(1)_ = 8.321^*^, *Φ* = 0.045), and grade 5 (*χ*^2^_(1)_ = 4.860^*^, *Φ* = 0.034). The proportion of students in grade 1 was significantly lower than grade 3 (*χ*^2^_(1)_ = 5.960^*^, *Φ* = 0.037). The proportion of students in grade 2 was significantly lower than grade 3 (*χ*^2^_(1)_ = 72.867^**^, *Φ* = 0.132), grade 4 (*χ*^2^_(1)_ = 9.870^*^, *Φ* = 0.050), and grade 5 (*χ*^2^_(1)_ = 15.746^**^, *Φ* = 0.062). The proportion of students in grade 3 was significantly higher than grade 4 (*χ*^2^_(1)_ = 27.003^**^, *Φ* = 0.082) and grade 5 (*χ*^2^_(1)_ = 21.032^**^, *Φ* = 0.071). There was no significant difference in the proportion between grade 4 and grade 5 (*χ*^2^_(1)_ = 0.533, *Φ* = 0.012). This result showed that the proportion of overcontrolled students was highest in grade 3 and lowest in grade 2. There was no significant difference in the proportion of boys and girls (*χ*^2^_(1)_ = 0.907, *Φ* = 0.010).

Among the resilient primary school students, there were significant differences between different grades (*χ*^2^_(4)_ = 138.021^**^, *Φ* = 0.115). The proportion of students in grade 1 was significantly lower than grade 2 (*χ*^2^_(1)_ = 4.975^*^, *Φ* = 0.034). The proportion of students in grade 1 was significantly higher than grade 3 (*χ*^2^_(1)_ = 46.181^**^, *Φ* = 0.103), grade 4 (*χ*^2^_(1)_ = 5.947^*^, *Φ* = 0.038), and grade 5 (*χ*^2^_(1)_ = 55.306^**^, *Φ* = 0.114). The proportion of students in grade 2 was significantly higher than grade 3 (*χ*^2^_(1)_ = 78.852^**^, *Φ* = 0.137), grade 4 (*χ*^2^_(1)_ = 20.578^**^, *Φ* = 0.072), and grade 5 (*χ*^2^_(1)_ = 90.245^**^, *Φ* = 0.147). The proportion of students in grade 3 was significantly lower than grade 4 (*χ*^2^_(1)_ = 17.140^**^, *Φ* = 0.065). There was no significant difference in the proportion between grade 3 and grade 5 (*χ*^2^_(1)_ = 0.452, *Φ* = 0.010). The proportion of students in grade 4 was significantly higher than grade 5 (*χ*^2^_(1)_ = 22.820^**^, *Φ* = 0.076). This result showed that as the grade level increased, the proportion of resilient students in primary schools generally showed a downward trend. The proportion of resilient students was highest in grade 2 and lowest in grade 5. The result showed that the proportion of resilient primary school boys was significantly lower than that of the girls (*χ*^2^_(1)_ = 87.235^**^, *Φ* = 0.092). There was a significant gender difference in resilient primary school students.

## Discussion

### Classification and Personality Characteristics of Students’ Personality Types

We divided the primary school students into three personality types using LPA. The types, resilient, overcontrolled, and undercontrolled, were consistent with previous research results (Donnellan and Robins, 2010; Meeus et al., 2011; Rosenström and Jokela, 2017). We used five personality dimensions as outcome variables and type as grouping variable to do a one-way analysis of variance; resilients had the highest scores, undercontrollers had the lowest scores on all of the five personality dimensions, and overcontrollers’ scores were between the other two types, with lower emotional stability, consistent with previous research results (Zentner and Shiner, 2012; Chen, 2019; Zou et al., 2019). The overcontrolled type represented the majority of the total sample. This finding was similar to the results of research by Ma (2016), who also found that the overcontrolled group accounted for the majority in China. This may reflect the limitations of Chinese social norms (Xie et al., 2016). Chang et al. (2011) considered students in China to be rule-abiding in their behavior patterns and encouraged to be compliant by Chinese teachers, which reflects the high requirements and expectations of self-control for students in China’s primary education system on the whole. This would explain why most Chinese students would fall in the overcontrolled group. The overcontrolled children were described by teachers as prosocial, well-liked by children and adults, and obedient and not as aggressive, self-assertive, and competitive. Resilients scored high on all five dimensions. The resilient children were described by self-confidence, independence, verbal fluency, and an ability to concentrate on tasks (Robins et al., 1996). Chinese teachers do not have a positive attitude toward all these characteristics. Compared with independent thinkers, teachers actually prefer overcontrolled students. Therefore, the teacher’s assessment of students may have observer bias. In our study, we took a multi-informant approach to reduce reporter bias in measurement of personality. Undercontrollers scored low on all five dimensions. The undercontrolled children were described by impulsivity, disobedience, stubbornness, and physical activity (Robins et al., 1996). Some of these characteristics might be considered advantages in the United States (e.g., being stubborn, physically active, uninhibited, and disobedient).

### Developmental Characteristics of Students’ Personality Types

We found that as the grade level increased, the proportion of undercontrolled students in primary schools generally showed an upward trend. Specifically, this type showed an upward trend from grade 1 to grade 2, a flat trend from grade 2 to grade 3, and an upward trend from grade 3 to grade 5. The proportion of resilient students in primary schools generally showed a downward trend. The proportion of resilient students was highest in grade 2 and lowest in grade 5. In the whole primary school stage, overcontrolled students always made up the majority. Individual physical and mental development and the learning atmosphere change as children progress through the grades, and those changes are reflected in the gradual decrease in the scores of certain personality dimensions (Galambos and Costigan, 2003). This is especially true for the non-adaptive undercontrolled primary school students. At the beginning of formal nine-year compulsory education, primary school students are in a transition period; they have not yet adapted to the changes in the learning environment, so their extraversion, openness, and emotional stability are on a downward trend (Soto et al., 2011; Van den Akker et al., 2014). The results of our study imply that middle-grade pupils have basically adapted to school, and most of their personality dimensions reflect a period of steady development (Yang et al., 2016). Therefore, the proportion of undercontrolled primary school students is relatively stable in the third and fourth grades. The proportion of undercontrolled primary school students increases rapidly after fourth grade, however. Entering the upper grades, primary school students not only face increased learning pressure, restrained creativity, reduced activity time, and decreased activity levels, but also physical and psychological changes. These changes are reflected in the declining trend of agreeableness, intelligence, extraversion, and conscientiousness (Van den Akker et al., 2010, 2014; Soto et al., 2011). This leads to an increase in the proportion of undercontrolled students and a decrease in the proportion of resilient students. At this age, resilient students are more likely to become undercontrolled or overcontrolled (Chen, 2019).

The proportion of girls with the resilient personality type was significantly higher than that of boys; conversely, the proportion of boys with the undercontrolled personality type was significantly higher than that of girls. The overcontrolled personality type did not show significant gender differences. The gender difference in primary school students’ personality types is consistent with the results of previous studies (Asendorpf and Van Aken, 1999). Gender differences in personality are either due to physical differences or due to gender socialization in childhood (Van den Akker et al., 2014). Chinese social culture gives different behaviors and attitudes suitable for boys and girls. In the process of Chinese gender role socialization, girls are expected to show the gentleness, dignity, and virtue of a “lady” who adapts to the environment and exhibits self-control. Boys are expected to be “brave” and “fearless” and are encouraged to show impulsiveness, seek stimulus, and otherwise exhibit poor inhibition. Physiologically, girls secrete fewer male hormones than boys and then adopt more mature self-regulation methods when coping with stressful events. They usually show less impulsive and aggressive behaviors. Therefore, boys who cannot effectively restrain impulses and adapt to the environment are more inclined to become undercontrolled primary school students.

## Conclusion

Primary school students could be divided into three personality types: the resilient, the overcontrolled, and the undercontrolled. Resilients had the highest scores, and undercontrollers had the lowest scores on all of five personality dimensions. The overcontrollers’ scores on personality were between the other two types, with lower emotional stability. As the grade level increased, the proportion of undercontrolled students in primary schools generally showed an upward trend and reached the maximum in grade 5. The proportion of resilient students in primary schools generally showed a downward trend. The proportion of resilient students was highest in grade 2 and lowest in grade 5. The proportion of girls with the resilient personality type was significantly higher than that of boys; conversely, the proportion of boys with the undercontrolled personality type was significantly higher than that of girls. The overcontrolled personality type did not show significant gender differences.

## Data Availability Statement

The datasets for this manuscript are not publicly available because the topic is not finished. Requests to access the datasets should be directed to corresponding author.

## Ethics Statement

The studies involving human participants were reviewed and approved by Jilin University Ethics Committee. Written informed consent to participate in this study was provided by the participants’ legal guardian.

## Author Contributions

YY designed the experiment, prepared the materials, and performed the experiment. YY and YZ analyzed the data and wrote the manuscript. All authors contributed to the article and approved the submitted version.

## Conflict of Interest

The authors declare that the research was conducted in the absence of any commercial or financial relationships that could be construed as a potential conflict of interest.

## Publisher’s Note

All claims expressed in this article are solely those of the authors and do not necessarily represent those of their affiliated organizations, or those of the publisher, the editors and the reviewers. Any product that may be evaluated in this article, or claim that may be made by its manufacturer, is not guaranteed or endorsed by the publisher.
